# Supplementary figures and images for: Testing the reproducibility of ecological studies on insect behavior in a multi-laboratory setting identifies opportunities for improving experimental rigor
Source: PLoS Biol. 2025 Apr 22;23(4):e3003019. doi: 10.1371/journal.pbio.3003019 (PMC12013911; doi:10.1371/journal.pbio.3003019)

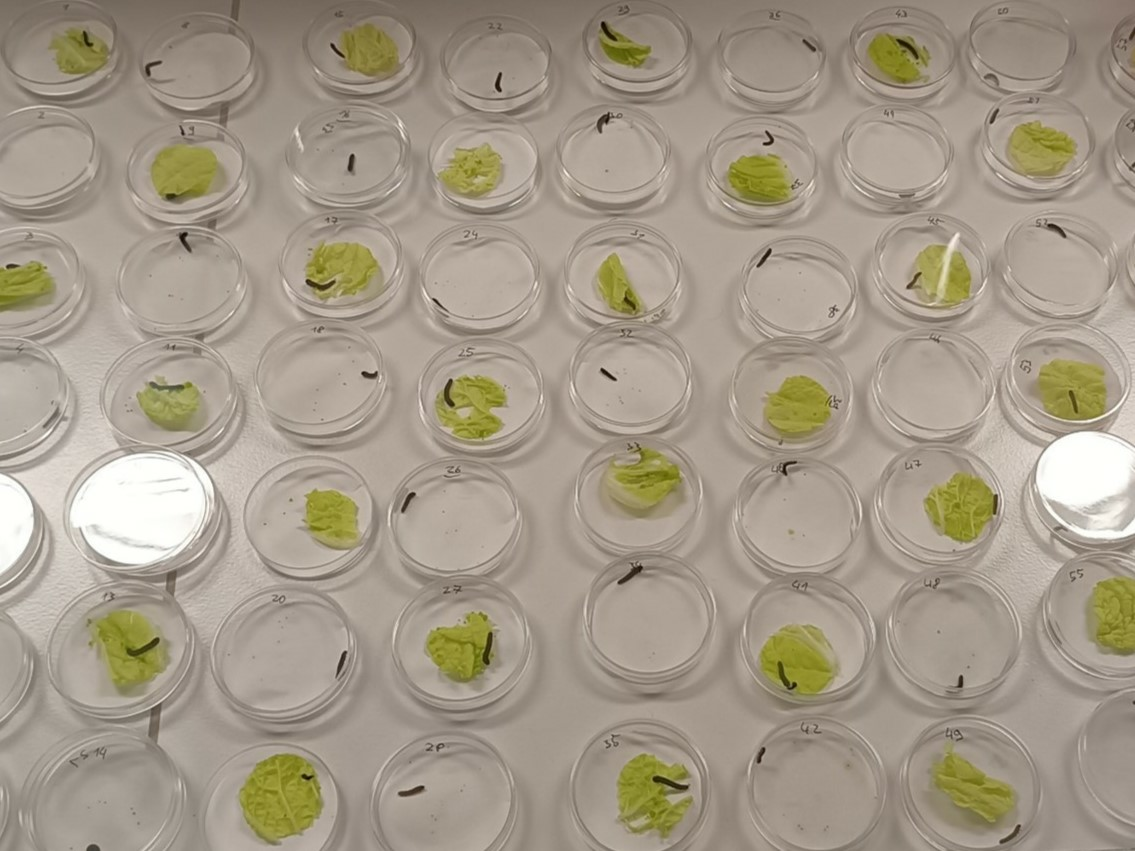

Supplement: S1 Fig — Photo credit: Maximilian Schurig. (TIF) [file pbio.3003019.s001.tif]

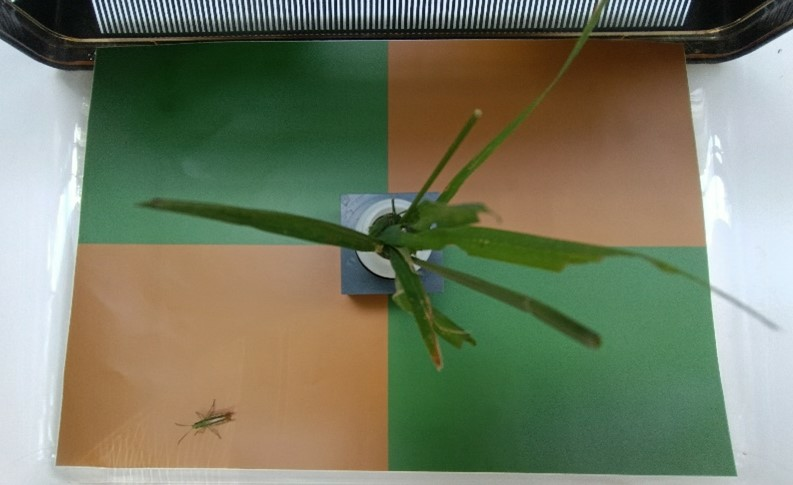

Supplement: S2 Fig — Photo credit: Maximilian Schurig. (TIF) [file pbio.3003019.s002.tif]

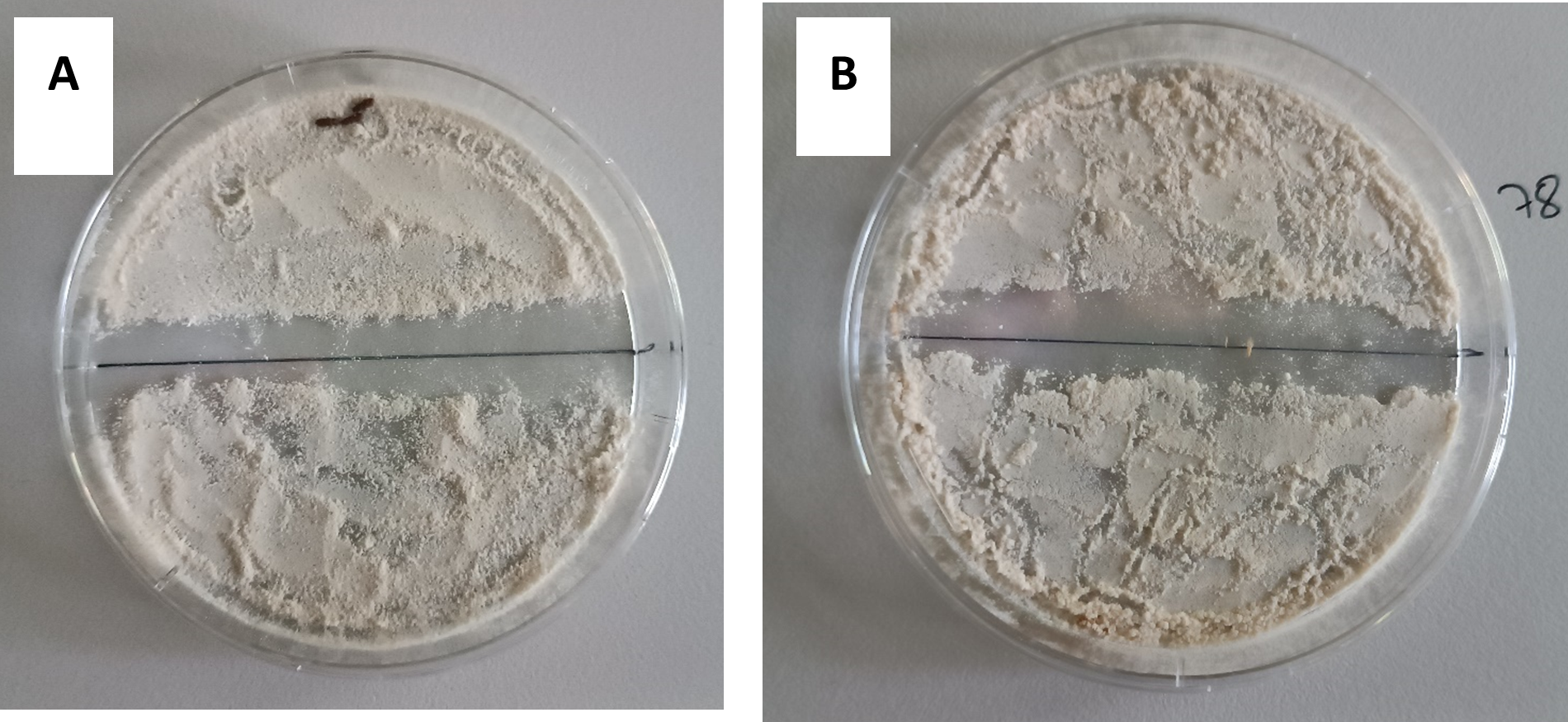

Supplement: S3 Fig — Photo was taken 24 h after the start of the experiment. Photo credit: Maximilian Schurig. (TIF) [file pbio.3003019.s003.tif]

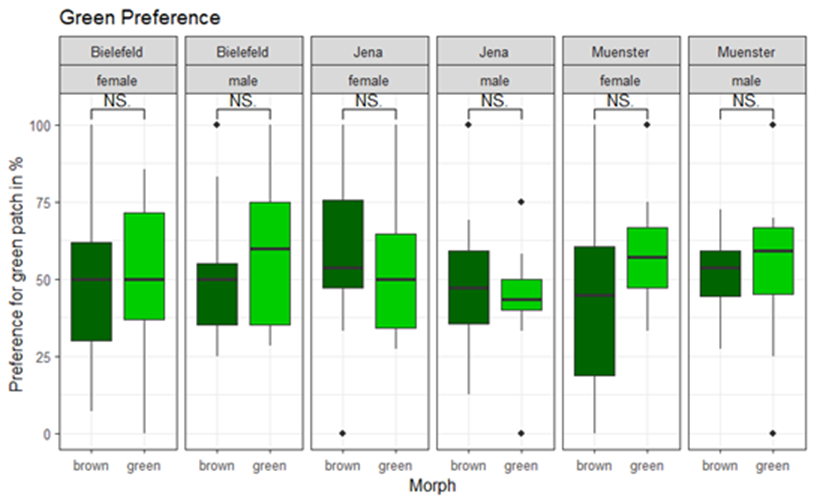

Supplement: S4 Fig — Data are presented as boxplots showing medians, 25% and 75% percentiles, and 5% and 95% percentiles. Statistics: Wilcoxon signed-rank test, two-sided, on the untransformed data *p < 0.05, **p < 0.01, ***p ≤ 0.001. The data and code needed to reproduce this Figure can be found in https://zenodo.org/records/14002690. The data summarized in the Figures can be found in S7 Table. (TIF) [file pbio.3003019.s004.tif]

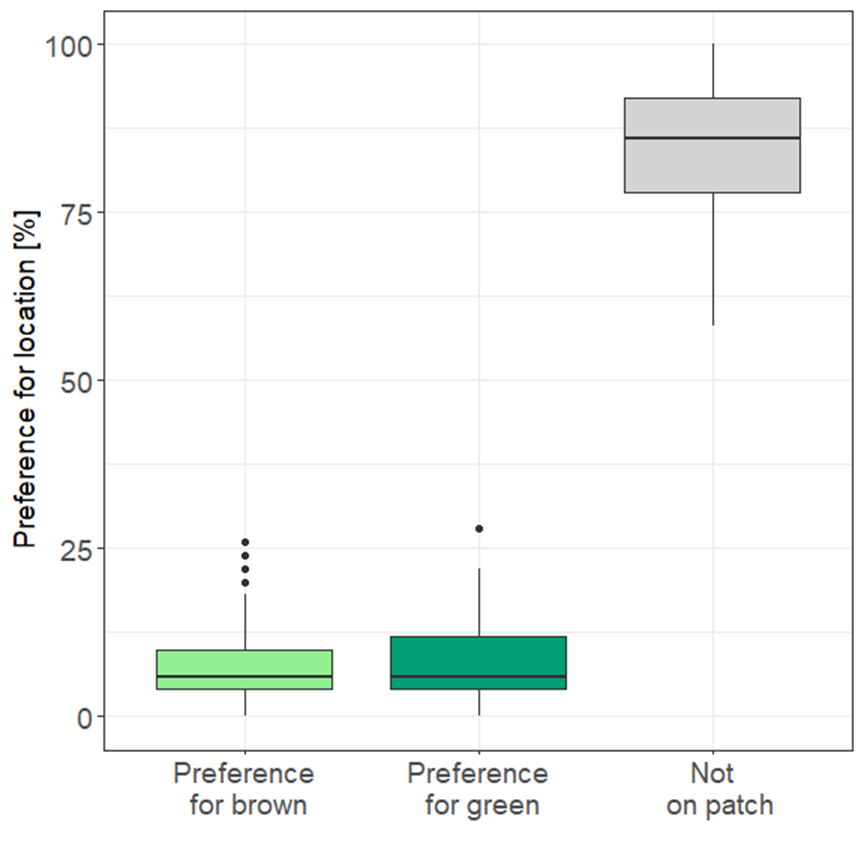

Supplement: S5 Fig — In 15.1% of those observations (1,329 instances), grasshoppers were sitting on the floor and could thus be assigned to one of the two patch colors. In the remaining 84.9% of observations (7,455 instances), grasshoppers were sitting on the cage walls, under the cage lids, or on the bundles of grass. Data are presented as boxplots showing medians, 25% and 75% percentiles, and 5% and 95% percentiles. The data and code needed to reproduce this Figure can be found in https://zenodo.org/records/14002690. The data summarized in the Figures can be found in S8 Table. (TIF) [file pbio.3003019.s005.tif]
