# Supplementary material for: Testing the reproducibility of ecological studies on insect behavior in a multi-laboratory setting identifies opportunities for improving experimental rigor
Source: PLoS Biol. 2025 Apr 22;23(4):e3003019. doi: 10.1371/journal.pbio.3003019 (PMC12013911; doi:10.1371/journal.pbio.3003019)
Supplement: S1 Table — (DOCX) [file pbio.3003019.s006.docx]

**Supplementary Table S1: Details on housing conditions, animals, preparation of materials and setup, experimental phase and experimenter specific characteristics for the *Athalia* experiment for each laboratory.**

| *Athalia* Bielefeld | *Athalia* Jena | *Athalia* Münster |
| --- | --- | --- |
| **Animals & Housing Conditions** | **Animals & Housing Conditions** | **Animals & Housing Conditions** |
| **Housing** | **Housing** | **Housing** |
| Light-dark cycle (e.g.,12/12; light on at: XY): 16/8 | Light-dark cycle (e.g.,12/12; light on at: XY): 16/8 | Light-dark cycle (e.g.,12/12; light on at: XY): 16/8 |
| Humidity: approximately 60% | Humidity: 70-80% | Humidity: approximately 60% |
| Temperature: room temperature (15-25°C) | Temperature: room temperature (15-25°C) | Temperature: room temperature (15-25°C) |
| Type of housing (e.g., incubator): mesh cage (60cm*60cm*60cm) | Type of housing (e.g., incubator): petri dish (5.5 cm ⌀) | Type of housing (e.g., incubator): mesh cage (60cm*60cm*60cm) |
| **Animals** | **Animals** | **Animals** |
| Maintenance container/petri dish etc. (measurements, shape, material etc.): mesh cage (60cm*60cm*60cm) | Maintenance container/petri dish etc. (measurements, shape, material etc.): petri dish (5.5 cm ⌀) | Maintenance container/petri dish etc. (measurements, shape, material etc.): mesh cage (60cm*60cm*60cm) |
| Housed in groups or alone: groups | Housed in groups or alone: alone | Housed in groups or alone: groups |
| Food type (e.g.,brand, or for Athalia: purchased from supermarket, self-grown etc.): chinese cabbage (Brassica rapa var. pekinensis) | Food type (e.g.,brand, or for Athalia: purchased from supermarket, self-grown etc.): chinese cabbage (organic) | Food type (e.g.,brand, or for Athalia: purchased from supermarket, self-grown etc.): chinese cabbage (Brassica rapa var. pekinensis) |
| Availability of food (e.g., ad libitum, restricted): ad libitum | Availability of food (e.g., ad libitum, restricted): ad libitum | Availability of food (e.g., ad libitum, restricted): ad libitum |
| Availability of water (e.g., ad libitum, restricted): No water but kept in petri dishes on moist filter paper | Availability of water (e.g., ad libitum, restricted): No water but kept in petri dishes on moist filter paper | Availability of water (e.g., ad libitum, restricted): No water but kept in petri dishes on moist filter paper |
| Cleaning routine (i.e. how often placed in new petri dish etc. e.g.,daily, weekly etc.): checked and cleaned as needed | Cleaning routine (i.e. how often placed in new petri dish etc. e.g.,daily, weekly etc.): cleaned every day | Cleaning routine (i.e. how often placed in new petri dish etc. e.g.,daily, weekly etc.): checked and cleaned as needed |
| Handling of animals (e.g., forceps): forceps | Handling of animals (e.g., forceps): forceps | Handling of animals (e.g., forceps): forceps |
| Date egglay: ~3/5/2023 | Date egglay: ~3/5/2023 | Date egglay: ~3/5/2023 |
| Arrival of the animals/parental generation (date): 16.05.2023 | Arrival of the animals/parental generation (date): 16.05.2023 | Arrival of the animals/parental generation (date): 16.05.2023 |
| Age at start experimental phase: Similar sized larvae chosen of ~3rd-4th instar | Age at start experimental phase: Similar sized larvae chosen of ~3rd-4th instar | Age at start experimental phase: Similar sized larvae chosen of ~3rd-4th instar |
| Age at end experimental phase: same age (~3rd-4th instar) | Age at end experimental phase: same age (~3rd-4th instar) | Age at end experimental phase: same age (~3rd-4th instar) |
| **Preparation of materials and setup** | **Preparation of materials and setup** | **Preparation of materials and setup** |
| Origin cabbage: grown from seeds in a greenhouse (20°C, 16L∶8D photoperiod, 70% relative humidity) | Origin cabbage: organic cabbage from Biomarkt Naturata Gera | Origin cabbage: bought at different grocery stores |
| **Experimental phase** | **Experimental phase** | **Experimental phase** |
| Date and time when experiments were performed (duration): 16.05.2023, 4h | Date and time when experiments were performed (duration): 14.05.2023, 9h | Date and time when experiments were performed (duration): 12.05.2023, 4h |
| Basic features apparatus/petri dish (measurements, shape, material etc.): petri dish (5.5 cm) | Basic features apparatus/petri dish (measurements, shape, material etc.): petri dish (5.5 cm) | Basic features apparatus/petri dish (measurements, shape, material etc.): petri dish (5.5 cm) |
| Separate room / same room as housing?: separate room | Separate room / same room as housing?: same room | Separate room / same room as housing?: separate room |
| Arrangement of treatment groups: alternating treatment replicates placed consecutively | Arrangement of treatment groups: alternating treatment replicates placed consecutively | Arrangement of treatment groups: alternating treatment replicates placed consecutively |
| Lighting condition (approx. lx?): room light (blinds shut) | Lighting condition (approx. lx?): room light (blinds shut) | Lighting condition (approx. lx?): room light (blinds open) |
| Camera type (e.g., brand): Basler ace acA 1300, Ahrensburg, Germany) | Camera type (e.g., brand): Smartphone camera | Camera type (e.g., brand): Logitec StreamCam |
| Interval of observations: one observation for max. 10 min | Interval of observations: one observation for max. 10 min | Interval of observations: one observation for max. 10 min |
| Tracking Software: Noldus Ethovision V7; Wageningen, NL | Tracking Software: ANY-maze | Tracking Software: ANY-maze |
| Temperature during experiment: ~20°C | Temperature during experiment: ~20°C | Temperature during experiment: ~20°C |
| **Experimenter specific characteristics** | **Experimenter specific characteristics** | **Experimenter specific characteristics** |
| Number of experimenters: 2 | Number of experimenters: 1 | Number of experimenters: 2 |
| Sex: male, female | Sex: male | Sex: male, female |
| Age (years): 30, 31 | Age (years): 22 | Age (years): 31, 42 |
| Experience in working with insects (e.g., no prior experience, years of experience etc.): 1 previous experiment for one experimenter, 3 years prior experience for second experimenter | Experience in working with insects (e.g., no prior experience, years of experience etc.): no previous experience | Experience in working with insects (e.g., no prior experience, years of experience etc.): no prior experience for one experimenter, 12 years prior experience with insects for second experimenter |
| Experience in the specific test paradigm (e.g., no prior experience, years of experience etc.): no prior experience for one experimenter, prior experience for second experimenter | Experience in the specific test paradigm (e.g., no prior experience, years of experience etc.): no prior experience | Experience in the specific test paradigm (e.g., no prior experience, years of experience etc.): no prior experience |
