# Supplementary material for: Testing the reproducibility of ecological studies on insect behavior in a multi-laboratory setting identifies opportunities for improving experimental rigor
Source: PLoS Biol. 2025 Apr 22;23(4):e3003019. doi: 10.1371/journal.pbio.3003019 (PMC12013911; doi:10.1371/journal.pbio.3003019)
Supplement: S2 Table — (DOCX) [file pbio.3003019.s007.docx]

**Supplementary Table S2: Details on housing conditions, animals, preparation of materials and setup, experimental phase and experimenter specific characteristics for the *Pseudochorthippus* experiment for each laboratory.**

| *Pseudochorthippus* Bielefeld | *Pseudochorthippus* Jena | *Pseudochorthippus* Münster |
| --- | --- | --- |
| **Animals & Housing Conditions** | **Animals & Housing Conditions** | **Animals & Housing Conditions** |
| **Housing** | **Housing** | **Housing** |
| Light-dark cycle (e.g.,12/12; light on at: XY): Collected from field | Light-dark cycle (e.g.,12/12; light on at: XY): 16/8 | Light-dark cycle (e.g.,12/12; light on at: XY): Collected from field |
| Humidity: Collected from field | Humidity: 70% | Humidity: Collected from field |
| Temperature: Collected from field | Temperature: 25-35°C | Temperature: Collected from field |
| Type of housing (e.g., incubator): Collected from field | Type of housing (e.g., incubator): housing cages | Type of housing (e.g., incubator): Collected from field |
| **Animals** | **Animals** | **Animals** |
| Maintenance container/petri dish etc. (measurements, shape, material etc.): small cylindrical vial | Maintenance container/petri dish etc. (measurements, shape, material etc.): housing cages | Maintenance container/petri dish etc. (measurements, shape, material etc.): small cylindrical vial |
| Housed in groups or alone: pairs (two individuals of the same morph and sex in a vial) | Housed in groups or alone: alone | Housed in groups or alone: pairs (two individuals of the same morph and sex in a vial) |
| Food type: collected from field so previous diet unknown, but during experiment they were fed on *ad libitum* grass collected from near Bielefeld University | Food type: grass collected from a field in Jena | Food type: collected from field so previous diet unknown, but during experiment they were fed on *ad libitum* grass collected behind the institute (Huefferstr.1, 48147 Muenster) |
| Availability of food (e.g., *ad libitum*, restricted): collected from field so previous diet unknown, but during experiment they were fed on ad libitum grass collected from near Bielefeld University | Availability of food (e.g., ad libitum, restricted): collected from field so previous diet unknown, but during experiment they were fed on ad libitum grass collected from near Bielefeld University | Availability of food (e.g., *ad libitum*, restricted): collected from field so previous diet unknown, but during experiment they were fed on ad libitum grass collected behind the institute (Huefferstr.1, 48147 Muenster) |
| Availability of water (e.g., *ad libitum*, restricted): collected from field so previous diet unknown, but during experiment they were fed on ad libitum grass collected from near Bielefeld University | Availability of water (e.g., ad libitum, restricted): fed on ad libitum grass collected Jena | Availability of water (e.g., *ad libitum*, restricted): ad libitum water |
| Cleaning routine (i.e., how often placed in new petri dish etc. e.g., daily, weekly etc.): used only during experiment | Cleaning routine (i.e., how often placed in new petri dish etc. e.g., daily, weekly etc.): used only during experiment | Cleaning routine (i.e., how often placed in new petri dish etc. e.g., daily, weekly etc.): used only during experiment |
| Handling of animals (e.g., forceps): no manual handling, opened the vial in the cage directly allowing one individual per cage | Handling of animals (e.g., forceps): no manual handling, opened the vial in the cage directly allowing one individual per cage | Handling of animals (e.g., forceps): no manual handling, opened the vial in the cage directly allowing one individual per cage |
| Arrival of the animals/parental generation (date): 29.06.2023 | Arrival of the animals/parental generation (date): 29.06.2023 | Arrival of the animals/parental generation (date): 29.06.2023 |
| Age at start experimental phase: age uncertain as adults collected from field | Age at start experimental phase: age uncertain as adults collected from field | Age at start experimental phase: age uncertain as adults collected from field |
| Age at end experimental phase: age uncertain as adults collected from field | Age at end experimental phase: age uncertain as adults collected from field | Age at end experimental phase: age uncertain as adults collected from field |
| **Preparation of materials and setup** | **Preparation of materials and setup** | **Preparation of materials and setup** |
| Origin grass: from outside the Bielefeld university (near 52°02&apos;13.6"N 8°29&apos;24.7"E) | Origin grass: from outside of Jena | Origin grass: behind the institute (Huefferstr.1, 48147 Muenster) |
| **Experimental phase** | **Experimental phase** | **Experimental phase** |
| Date and time when experiments were performed (duration): from 30.06.2023 to 07.07.2023 (7 days) | Date and time when experiments were performed (duration): from 1.07.2023 to 14.07.2023 (14 days) | Date and time when experiments were performed (duration): from 30.06.2023 to 07.07.2023 (7 days) |
| Basic features apparatus/petri dish (measurements, shape, material etc.): housing cage (Terra Exotica Faunabox, 37 cm × 22 cm × 24.5 cm) | Basic features apparatus/petri dish (measurements, shape, material etc.): housing cage (Terra Exotica Faunabox, 37 cm × 22 cm × 24.5 cm) | Basic features apparatus/petri dish (measurements, shape, material etc.): housing cage (Terra Exotica Faunabox, 37 cm × 22 cm × 24.5 cm) |
| Separate room / same room as housing?:  separate room | Separate room / same room as housing?:  same room | Separate room / same room as housing?:  same room |
| Arrangement of treatment groups: 1 individual per cage, located same sex individuals near each other but of alternating treatment | Arrangement of treatment groups: 1 individual per cage, located same sex individuals near each other but of alternating treatment | Arrangement of treatment groups: 1 individual per cage, located same sex individuals near each other but of alternating treatment |
| Lighting condition (approx. lx?): room lightning + natural daylight (cages were placed near windows) | Lighting condition (approx. lx?): room lightning + natural daylight (cages were placed near windows) | Lighting condition (approx. lx?): natural daylight (cages were in a glass house, which had significant shade from trees) |
| Camera type (e.g., brand): Not used | Camera type (e.g., brand): Not used | Camera type (e.g., brand): Not used |
| Interval of observations: 1h | Interval of observations: 1h | Interval of observations: 1h |
| Tracking Software: Not used | Tracking Software: Not used | Tracking Software: Not used |
| Temperature during experiment: room temperature | Temperature during experiment: 25°C | Temperature during experiment: 20-24C |
| Notes: they were housed briefly overnight in a cold room (4°C) | Notes: | Notes: |
| **Experimenter specific characteristics** | **Experimenter specific characteristics** | **Experimenter specific characteristics** |
| Number of experimenters: 1 | Number of experimenters: 1 | Number of experimenters: 1 |
| Sex: male | Sex: male | Sex: male |
| Age (years): 30 | Age (years): 23 | Age (years): 31 |
| Experience in working with insects (e.g., no prior experience, years of experience etc.): 2 previous experiments | Experience in working with insects (e.g., no prior experience, years of experience etc.): no previous experience | Experience in working with insects (e.g., no prior experience, years of experience etc.): 2 prior experiments |
| Experience in the specific test paradigm (e.g., no prior experience, years of experience etc.): no prior experience | Experience in the specific test paradigm (e.g., no prior experience, years of experience etc.): no prior experience | Experience in the specific test paradigm (e.g., no prior experience, years of experience etc.): no prior experience |
