# Supplementary material for: Testing the reproducibility of ecological studies on insect behavior in a multi-laboratory setting identifies opportunities for improving experimental rigor
Source: PLoS Biol. 2025 Apr 22;23(4):e3003019. doi: 10.1371/journal.pbio.3003019 (PMC12013911; doi:10.1371/journal.pbio.3003019)
Supplement: S3 Table — (DOCX) [file pbio.3003019.s008.docx]

**Supplementary Table S3: Details on housing conditions, animals, preparation of materials and setup, experimental phase and experimenter specific characteristics for the *Tribolium* experiment for each laboratory.**

| *Tribolium* Bielefeld | *Tribolium* Jena | *Tribolium* Münster |
| --- | --- | --- |
| **Animals & Housing Conditions** | **Animals & Housing Conditions** | **Animals & Housing Conditions** |
| **Housing** | **Housing** | **Housing** |
| Light-dark cycle (e.g.,12/12; light on at: XY): 12/12 | Light-dark cycle (e.g.,12/12; light on at: XY): always dark | Light-dark cycle 12h/12h, lights on @8am |
| Humidity: 60-65% | Humidity: 70-80% | Humidity: 60% |
| Temperature: 30°C | Temperature: 20 – 30°C | Temperature: 30°C |
| Type of housing: incubator | Type of housing: incubator | Type of housing: incubator |
| **Animals** | **Animals** | **Animals** |
| Maintenance container: Glass jars | Maintenance container: Glass jars | Maintenance container: Glass jars (1L) |
| Housed in groups or alone: groups | Housed in groups or alone: groups | Housed in groups |
| Food type: organic wheat flour (Type 550) from DM drugstore and 5% brewer&apos;s yeast (Münster lab provided) | Food type: organic wheat flour (Type 550) from DM drugstore and 5% brewer&apos;s yeast (Münster lab provided) | Food type: organic wheat flour (Type 550) from DM drugstore and 5% brewer&apos;s yeast (heat inactivated at 75C for 24 hours) |
| Availability of food (e.g., *ad libitum*, restricted): ad libitum | Availability of food (e.g., ad libitum, restricted): ad libitum | Availability of food (e.g., *ad libitum*, restricted): ad libitum |
| Availability of water (e.g., *ad libitum*, restricted): no water | Availability of water (e.g., ad libitum, restricted): no water | Availability of water (e.g., *ad libitum*, restricted): no water |
| Cleaning routine (i.e., how often placed in new petri dish etc. e.g., daily, weekly etc.): experimental generation eggs laid in new jar with fresh flour. For larvae, they were collected directly from jar. For adults, pupae were sexed and 5 female pupae put in 1 petri dish and then used for experiment 4 days after eclosion. | Cleaning routine (i.e., how often placed in new petri dish etc. e.g., daily, weekly etc.): exchange of flour every 2 weeks | Cleaning routine (i.e., how often placed in new petri dish etc. e.g., daily, weekly etc.): experimental generation eggs laid in new jar with fresh flour. For larvae, they were collected directly from jar. For adults, pupae were sexed and individualized in 96 well plates |
| Handling of animals (e.g.,forceps): paper strips and sieves (700um) | Handling of animals (e.g.,forceps): paper strips and sieves (700um) | Handling of animals (e.g.,forceps): soft forceps, paper strips and sieves (700um) |
| Date egglay: For larvae, eggs laid on 24/5/2023 and 25/5/2023. For adults, eggs laid on 9/5/2023 and 10/5/2023. | Date egglay: For larvae, eggs laid on 30/4/2024 and 05/5/2023. For adults, eggs laid on 16/4/2024 and 21/04/2024. | Date egglay: April and May 2023 |
| Arrival of the animals/parental generation (date): ~11/03/2023 | Arrival of the animals/parental generation (date): 17/03/2024 | Arrival of the animals/parental generation (date): ~11/03/2023 |
| Age at start experimental phase: ~14 days for larvae, ~4 days for adults | Age at start experimental phase: ~14 days for larvae, ~4 days for adults | Age at start experimental phase: NA |
| Age at end experimental phase: ~14 days for larvae, ~4 days for adults | Age at end experimental phase: ~14 days for larvae, ~4 days for adults | Age at end experimental phase: ~14 days for larvae, ~4 days for adults |
| **Preparation of materials and setup** | **Preparation of materials and setup** | **Preparation of materials and setup** |
| Storage of flour: (e.g., storage at 75°C for at least 18h to kill bacteria and fungi: storage at 75°C for 24h | Storage of flour: (e.g., storage at 75°C for at least 18h to kill bacteria and fungi: storage at 80°C for 24h | Storage of flour: (e.g., storage at 75°C for at least 18h to kill bacteria and fungi: storage at 75°C for 24h |
| Preparation flour (e.g., sieve with a 720um sieve before experiment): sieve with a 700um before experiment | Preparation flour (e.g., sieve with a 720um sieve before experiment): sieve with a 700um before experiment | Preparation flour (e.g., sieve with a 720um sieve before experiment): sieve with a 700um before experiment |
| Type of flour: organic wheat flour (Type 550) from DM drugstore | Type of flour: organic wheat flour (Type 550) | Type of flour: organic wheat flour (Type 550) from DM drugstore |
| **Experimental phase** | **Experimental phase** | **Experimental phase** |
| Date and time when experiments were performed (duration): 08.06.2023 and 12.06.2023, 6 hours each experiment and then one observation after 24h | Date and time when experiments were performed (duration): 20.06.2024 and 24.06.2024, 8 hours each experiment and then one observation after 24h | Date and time when experiments were performed (duration): 31.05.2023 and 14.06.2023, 6 hours each experiment and then one observation after 24h |
| Basic features apparatus/petri dish (measurements, shape, material etc.): petri dish (diameter 90mm) | Basic features apparatus/petri dish (measurements, shape, material etc.): petri dish (diameter 90mm) | Basic features apparatus/petri dish (measurements, shape, material etc.): petri dish (diameter 90mm) |
| Separate room / same room as housing?: separate room | Separate room / same room as housing?: same room | Separate room / same room as housing?: separate room |
| Arrangement of treatment groups: alternating treatment replicates placed consecutively | Arrangement of treatment groups: alternating treatment replicates placed consecutively | Arrangement of treatment groups: alternating treatment replicates placed consecutively |
| Lighting condition (approx. lx?): room light (blinds shut) | Lighting condition (approx. lx?): room light (blinds shut) | Lighting condition (approx. lx?): room light (blinds shut) |
| Camera type (e.g.,brand): Not used | Camera type (e.g.,brand): Not used | Camera type (e.g.,brand): Not used |
| Interval of observations: 30 min | Interval of observations: 30 min | Interval of observations: 30 min |
| Tracking Software: Not used | Tracking Software: Not used | Tracking Software: Not used |
| Temperature during experiment: ~20°C | Temperature during experiment: ~30°C | Temperature during experiment: ~20°C |
| **Experimenter specific characteristics** | **Experimenter specific characteristics** | **Experimenter specific characteristics** |
| Number of experimenters: 2 | Number of experimenters: 1 | Number of experimenters: 2 |
| Sex: male, female | Sex: male | Sex: male, female |
| Age (years): 30, 31 | Age (years): 22 | Age (years): 31, 42 |
| Experience in working with insects (e.g., no prior experience, years of experience etc.): 1 previous experiment for one experimenter, 3 years prior experience for second experimenter | Experience in working with insects (e.g., no prior experience, years of experience etc.): no previous experience | Experience in working with insects (e.g., no prior experience, years of experience etc.): 1 previous experiment for one experimenter, 12 years prior experience for second experimenter with specific species |
| Experience in the specific test paradigm (e.g., no prior experience, years of experience etc.): no prior experience | Experience in the specific test paradigm (e.g., no prior experience, years of experience etc.): no prior experience | Experience in the specific test paradigm (e.g., no prior experience, years of experience etc.): no prior experience |
