# Supplementary material for: Testing the reproducibility of ecological studies on insect behavior in a multi-laboratory setting identifies opportunities for improving experimental rigor
Source: PLoS Biol. 2025 Apr 22;23(4):e3003019. doi: 10.1371/journal.pbio.3003019 (PMC12013911; doi:10.1371/journal.pbio.3003019)
Supplement: S4 Table — (DOCX) [file pbio.3003019.s009.docx]

**Supplementary Table S4A: Descriptive Statistics of the outcome measure “PCI duration” [sec] in the *Athalia* experiment for each group across all labs.**

| **Group** | **Mean (SD)** | **Median** | **Max** | **Min** | **Sample size** |
| --- | --- | --- | --- | --- | --- |
| Control | 266.3 (234.6) | 181 | 600 | 0 | 90 |
| Treatment | 78.9 (142.8) | 25 | 600 | 0 | 90 |

**Supplementary Table S4B: Descriptive Statistics of the outcome measure “PCI duration” [sec] in the *Athalia* experiment within each lab and group.**

| **Lab** | **Group** | **Mean (SD)** | **Median** | **Max** | **Min** | **Sample size** |
| --- | --- | --- | --- | --- | --- | --- |
| Bielefeld | Control | 424.63(182.2) | 460.5 | 600 | 75 | 30 |
|  | Treatment | 48.8 (108.2) | 23.5 | 600 | 6 | 30 |
| Jena | Control | 174.0 (226.4) | 60.0 | 600 | 0 | 30 |
|  | Treatment | 97.0 (157.1) | 33.5 | 600 | 0 | 30 |
| Muenster | Control | 200.4 (212.7) | 129.0 | 600 | 0 | 30 |
|  | Treatment | 91.4 (157.4) | 18.0 | 600 | 0 | 30 |

**Supplementary Table S4C: Descriptive Statistics of the outcome measure “distance moved” [cm] in the Athalia experiment** **for each group across all labs.**

| **Group** | **Mean (SD)** | **Median** | **Max** | **Min** | **Sample size** |
| --- | --- | --- | --- | --- | --- |
| Control | 25.9 (27.8) | 14.5 | 131.9 | 0 | 90 |
| Treatment | 64.8 (31.8) | 67.2 | 143.8 | 0 | 90 |

**Supplementary Table S4D: Descriptive Statistics of the outcome measure “distance moved” [cm] in the Athalia experiment within each lab and group.**

| **Lab** | **Group** | **Mean (SD)** | **Median** | **Max** | **Min** | **Sample size** |
| --- | --- | --- | --- | --- | --- | --- |
| Bielefeld | Control | 14.09 (14.30) | 9.72 | 67.37 | 2.03 | 30 |
|  | Treatment | 58.12 (25.42) | 66.72 | 109.44 | 4.44 | 30 |
| Jena | Control | 37.10 (24.05) | 36.91 | 84.00 | 0.00 | 30 |
|  | Treatment | 62.87 (36.41) | 66.88 | 120.00 | 0.00 | 30 |
| Muenster | Control | 27.86 (36.29) | 7.05 | 131.90 | 0.00 | 30 |
|  | Treatment | 73.47 (31.55) | 73.35 | 143.80 | 12.20 | 30 |
