# Supplementary material for: Testing the reproducibility of ecological studies on insect behavior in a multi-laboratory setting identifies opportunities for improving experimental rigor
Source: PLoS Biol. 2025 Apr 22;23(4):e3003019. doi: 10.1371/journal.pbio.3003019 (PMC12013911; doi:10.1371/journal.pbio.3003019)
Supplement: S5 Table — (DOCX) [file pbio.3003019.s010.docx]

| **Morph** | **Mean (SD)** | **Median** | **Max** | **Min** | **Sample size** |
| --- | --- | --- | --- | --- | --- |
| brown | 50.29 (24.58) | 50.00 | 0.00 | 100.00 | 90 |
| green | 53.47 (21.89) | 52.27 | 0.00 | 100.00 | 88 |

**Supplementary Table S5A: Descriptive Statistics of the outcome measure “substrate choice” as percent of individuals on green patch [%] in the *Pseudochorthippus* experiment for each morph type across all labs.**

**Supplementary Table S5B: Descriptive Statistics of the outcome measure “substrate choice” as percent of individuals on green patch [%] in the Pseudochorthippus experiment within each lab and morph type.**

| **Lab** | **Morph** | **Mean (SD)** | **Median** | **Max** | **Min** | **Sample size** |
| --- | --- | --- | --- | --- | --- | --- |
| Bielefeld | brown | 49.67 (22.89) | 50.00 | 7.14 | 100.00 | 30 |
|  | green | 54.15 (24.09) | 60.00 | 0.00 | 100.00 | 28 |
| Jena | brown | 53.81 (26.22) | 50.00 | 0.00 | 100.00 | 29 |
|  | green | 48.98 (20.33) | 44.44 | 0.00 | 100.00 | 29 |
| Muenster | brown | 47.59 (25.00) | 50.00 | 0.00 | 100.00 | 31 |
|  | green | 57.06 (21.19) | 57.14 | 0.00 | 100.00 | 31 |
