# Supplementary material for: Testing the reproducibility of ecological studies on insect behavior in a multi-laboratory setting identifies opportunities for improving experimental rigor
Source: PLoS Biol. 2025 Apr 22;23(4):e3003019. doi: 10.1371/journal.pbio.3003019 (PMC12013911; doi:10.1371/journal.pbio.3003019)
Supplement: S6 Table — (DOCX) [file pbio.3003019.s011.docx]

**Supplementary Table S6A: Descriptive Statistics of the outcome measure “niche choice” as percent of individuals in Drak-flour [%] in the Tribolium experiment for each group across all labs.**

| **Group** | **Mean (SD)** | **Median** | **Min** | **Max** | **Sample size** |
| --- | --- | --- | --- | --- | --- |
| Adult | 56.8 (29.9) | 58.3 | 0 | 100 | 321 |
| Larvae | 53.3 (19.1) | 53.8 | 0 | 100 | 320 |

**Supplementary Table S6B: Descriptive Statistics of the outcome measure “niche choice” as percent of individuals in Drak-flour [%] in the Tribolium experiment within each lab and group.**

| **Lab** | **Group** | **Mean (SD)** | **Median** | **Min** | **Max** | **Sample size** |
| --- | --- | --- | --- | --- | --- | --- |
| Bielefeld | Adult | 53.4 (28.9) | 55.00 | 0.00 | 100.00 | 81 |
|  | Larvae | 45.6 (21.0) | 43.60 | 5.13 | 100.00 | 81 |
| Jena | Adult | 52.4 (20.1) | 50.00 | 5.00 | 100.00 | 120 |
|  | Larvae | 48.7 (12.2) | 51.30 | 5.13 | 71.8 | 120 |
| Muenster | Adult | 63.5 (37.0) | 78.60 | 0.00 | 100.00 | 120 |
|  | Larvae | 62.9 (19.5) | 66.70 | 0.00 | 100.00 | 120 |
