# Supplementary material for: Testing the reproducibility of ecological studies on insect behavior in a multi-laboratory setting identifies opportunities for improving experimental rigor
Source: PLoS Biol. 2025 Apr 22;23(4):e3003019. doi: 10.1371/journal.pbio.3003019 (PMC12013911; doi:10.1371/journal.pbio.3003019)
Supplement: S7 Table — (DOCX) [file pbio.3003019.s012.docx]

**Supplementary Table S7: Descriptive Statistics of the outcome measure “substrate choice” as percent of individuals on green patch [%] in the *Pseudochorthippus* experiment within each lab, sex and morph type.**

| **Lab** | **Sex** | **Morph** | **Mean (SD)** | **Median** | **Min** | **Max** | **Sample size** |
| --- | --- | --- | --- | --- | --- | --- | --- |
| Bielefeld | female | brown | 48.45 (24.89) | 50.00 | 7.14 | 100.00 | 15 |
|  |  | green | 49.88 (27.15) | 50.00 | 0.00 | 85.71 | 13 |
|  | male | brown | 50.89 (21.50) | 50.00 | 25.00 | 100.00 | 15 |
|  |  | green | 57.85 (21.34) | 60.00 | 28.57 | 100.00 | 15 |
| Jena | female | brown | 60.34 (27.47) | 53.85 | 0.00 | 100.00 | 15 |
|  |  | green | 54.37 (22.74) | 50.00 | 27.27 | 100.00 | 15 |
|  | male | brown | 46.81 (23.80) | 47.22 | 12.50 | 100.00 | 14 |
|  |  | green | 43.20 (16.23) | 43.65 | 0.00 | 75.00 | 14 |
| Muenster | female | brown | 42.74 (32.66) | 45.00 | 0.00 | 100.00 | 16 |
|  |  | green | 59.95 (19.99) | 57.14 | 33.33 | 100.00 | 15 |
|  | male | brown | 52.76 (11.85) | 53.85 | 27.27 | 72.73 | 15 |
|  |  | green | 54.35 (22.56) | 59.34 | 0.00 | 100.00 | 16 |
