# Supplementary material for: Testing the reproducibility of ecological studies on insect behavior in a multi-laboratory setting identifies opportunities for improving experimental rigor
Source: PLoS Biol. 2025 Apr 22;23(4):e3003019. doi: 10.1371/journal.pbio.3003019 (PMC12013911; doi:10.1371/journal.pbio.3003019)
Supplement: S8 Table — (DOCX) [file pbio.3003019.s013.docx]

**Supplementary Table S8: Descriptive Statistics of the recorded location [%] across all individuals in the *Pseudochorthippus* experiment across all labs.**

| **Location** | **Mean (SD)** | **Median** | **Min** | **Max** | **Sample size** |
| --- | --- | --- | --- | --- | --- |
| Brown patch | 7.27 (5.62) | 6.00 | 0.00 | 26.00 | 185 |
| Green patch | 7.64 (5.41) | 6.00 | 0.00 | 28.00 | 185 |
| Not on patch | 85.09 (9.3) | 86.00 | 58.00 | 100.00 | 185 |
