# Supplementary material for: Testing the reproducibility of ecological studies on insect behavior in a multi-laboratory setting identifies opportunities for improving experimental rigor
Source: PLoS Biol. 2025 Apr 22;23(4):e3003019. doi: 10.1371/journal.pbio.3003019 (PMC12013911; doi:10.1371/journal.pbio.3003019)
Supplement: Attachment — Submitted filename: Response to Reviewers.docx [file pbio.3003019.s016.docx]

**Response to reviewers’ comments:**

**Reviewer #1:**

This study is an important addition to the growing body of work devoted to describing and

attempting to explain variability in results among empirical studies. These researchers

demonstrate, in studies of three very different species of insects, that observed behavioral

responses to apparently identical experiments sometimes differed substantially when

conducted in different labs. This is similar to results that have been observed in a number of

sets of laboratory rodent studies. This current study is useful in that it demonstrates this

phenomenon in a different group of organisms, and because it brings the work to the attention

of a different set of researchers - animal behavior researchers more closely tied to the

disciplines of evolutionary biology and ecology.

The study appears well designed and well executed, and the data analyses appear reasonable.

*Authors’ response:*

Thank you very much for the positive feedback!

I would be interested to read the authors' thoughts on the implications of their work for	understanding the substantial heterogeneity that has been demonstrated to exist among	published statistical effects in ecology and evolutionary biology (for instance, by Senior et al.	2016. Ecology, 97:3293-3299).

*Authors’ response:*

Thank you for pointing out this study! Senior et al. (2016) investigated the extent of heterogeneity in meta-analyses across various fields of ecology and evolutionary biology. They argue that in meta-analyses in which each effect size comes from a different species, inter-specific differences are likely to cause variation among effect sizes. While this in itself is interesting and highly plausible, it does not strictly apply to our work. Here, we conducted three single random-effect meta analyses, one for each type of experiment. Consequently our meta analyses do not incorporate effect sizes of different species, but only the effect sizes of the respective replicate experiments for the respective species. We would therefore prefer to not include these thoughts in the manuscript.

I have arranged my other comments by line number.

66 - "Attempts to replicate core scientific finding have produced mixed to rather discouraging	results (e.g., Ioannidis, 2005; Freedman et al., 2015; Baker, 2016),"

The "e.g.," here is misleading, because it implies that you are listing examples of studies that

attempted to replicate scientific findings. However, the studies you cited do not actually

attempt replications, themselves. The Baker paper surveyed researchers about the practices

and experiences, and the Ioannidis paper mostly presents a model and an argument. I don't

know the Freedman et al. paper. So, the solution would be to either cite papers that actually	assessed replicability, or to delete "e.g.,"

Also, I think the term 'core' overstates the case here. Are these findings that failed to

replicate actually at the core of science such that their failure to replicate brings down the

entire edifice?

*Authors’ response:*

We followed the suggestion and removed “e.g.” as well as “core”. It now reads (lines 66-69):

“Attempts to replicate scientific finding have produced mixed to rather discouraging results [3–5], raising concerns whether reproducibility is as high as is desirable and coining the term “reproducibility crisis” (e.g., [3,6,5,7])“

69 - "70% of researchers reported,"

I suggest editing to something like "70% of researchers who responded to a survey	reported…" since these surveys are not representative

*Authors’ response:*

We followed the suggested rewording (lines 69-70):

“Furthermore, more than 70% of researchers who responded to a survey reported having tried and failed to reproduce another scientist's experiments, and more than half admitted having failed to reproduce their own experiments [5].”

75 - (and throughout) - I encourage you to reconsider your widespread us of the term 'reproducibility' and to instead use 'replicability'. There is a strong trend in the growing metascience movement to converge on the use of the terms according to these definitions: (taken from <https://www.ncbi.nlm.nih.gov/books/NBK547546/>)

"… reproducibility is obtaining consistent results using the same input data; computational steps, methods, and code; and conditions of analysis. This definition is synonymous with "computational reproducibility," ….

Replicability is obtaining consistent results across studies aimed at answering the same scientific question, each of which has obtained its own data."

*Authors’ response:*

While we are aware of the inconsistent use and definitions of the terms “reproducibility” and “replicability”, we prefer to stick with the term “reproducibility”. This is for two reasons: 1) we are strongly building on (and referencing) the popular publication of Baker et al. (2016), who herself coined the term “reproducibilty crisis” and 2) we want to stay consistent with the previously published work of our authors. However, for clarity we added a definition in the introduction (lines 63-65):

“Reproducibility, i.e., the ability of a result to be replicated by an independent experiment in the same or different laboratory [1]; also referred to as replicability [2], is a cornerstone of any scientific method.”

86 - do you mean "guides" (plural)?

*Authors’ response:*

We rephrased this second part to improve understanding (lines 85-86):
“Consequently, researchers started to search for causes of poor reproducibility and ways out of the crisis”.

111 - You may wisht to consult and incorporate results in this paper:

Milcu et al. 2018 <https://doi.org/10.1038/s41559-017-0434-x>

This paper examines the issue in an ecological study using plant microcosms

*Authors’ response:*

We now include this study in the discussion regarding standardization and reproducibility (paragraph 4.1.1; lines 606 -607):

“Similarly, an ecological study on grass grown in microcosms showed that a controlled systematic increase in genetic variation reduced variation among laboratories [73].”

224,227 - The treatment is confusing. Four hours without food, and then 'after three hours of treatment'. So, does this mean 4+3 = 7 hours of starvation, or something else?

OK, I see that this is clarified later in the paragraph, but it would be useful to clarify it immediately.

*Authors’ response:*

We removed the mentioning of the “4 h” in the first sentence, so it does not cause any confusion, when the exact times are mentioned later in the paragraph (lines 224-226): “In the starvation treatment larvae had no access to cabagge leaves, while in the control treatment larvae were provided cabbage leaves *ad libitum* (see Supplementary Fig S1).”

344: the zenodo files are not publicly accessible, and so I cannot assess them

*Authors’ response:*

The Zenodo files (data as well as all code for analysis and plotting) are now publicly available:
https://zenodo.org/records/14002690

389: which type of effect size is this?

*Authors’ response:*

The overall treatment effect is based on the respective treatment effect sizes of each experiment (as the beta-estimates from the GLMMs) and standard errors of all laboratories. This is explained in lines 387-389:

“The meta-analytic overall effect size was estimated by a random-effect meta-analysis using the metafor-package (version 4.4-0; Viechtbauer, 2010) based on the individual treatment effect sizes and standard errors of all laboratories.”

422: "did not detect any differences… p = 0.14"

I encourage you to moderate you wording here. The means differed, it's just that the p-value did not cross your arbitrary threshold. I suggest "did not detect a statistically significant difference".

I notice that you cite Amrhein et al. in the discussion - their perspective is relevant here.

*Authors’ response:*

We followed the suggestion and rephrased the wording and also added a reference to the p-value discussion of the later chapter (lines 420-422):

„In contrast, the third laboratory did not detect a statistically significant difference between the starved and control larvae (Lab B: W = 549.5, p = 0.14; see Fig 3A; but please see critical discussion on the use of p-values in paragraph 4.1.3).”

Fig 3. Please identify the type of effect size.

Also, it seems that it could be valuable to also indicate on these graphs the effect sizes produced by the original published studies being replicated here.

*Authors’ response:*

We added a clarification on the effect size in the figure legend (lines 440-449):

“**Fig 3**. **Variation of treatment differences across the three replicate experiments for (A) the post-contact immobility (PCI) duration and (B) distance moved in larvae of *A. rosae*, (C) the substrate preference in the two colour morphs of *P. parallelus,* and (D) the niche preference in the two life stages of *T. castaneum*.** The black solid line reflects the null effect. The red dashed line and grey area indicate the overall absolute (abs) effect size and its corresponding 95% confidence interval (CI_95_). Dots and vertical dashed lines reflect the mean group differences (as estimated from the GLMMs) and corresponding CI_95_ of the three laboratories. The overall absolute effect size was estimated by a random-effect meta-analysis based on the individual treatment effect sizes and standard errors of all laboratories. The data and code needed to reproduce this Figure can be found in <https://zenodo.org/records/14002690.>”

And while we agree that directly comparing effect sizes with the original published studies would be interesting, differences in experimental details—particularly for *Tribolium* and *Pseudochorthippus*—prevent a meaningful comparison.

471: I think a word may be missing after "a"

*Authors’ response:*

Yes, thank you! We added the missing word (lines 480-481):

“In 33.4% of the observations (4,116 instances), adult beetles were on their feet, so that a preference could be recorded.”

479: "could not be reproduced" implies an effort to get the same result. Wouldn't it be more accurate to say "did not reproduce" since this does not imply an effort to get a particular result? (or actually, "did not replicate", see earlier comment)

*Authors’ response:*

Yes, this is a valid point. We rephrased as suggested (lines 489-490):

“The third laboratory did not replicate significant group differences (Lab B: W = 7699.5, p = 0.30). ”

Figure 5. The Y-axis is labeled as "preference for dark flour", but no where in the paper do you discuss "dark flour"

*Authors’ response:*

The “*Drak* flour” is the flour conditioned by beetles that received a knockdown treatment for the *Drak* gene. These beetles do not develop functioning stink glands and the flour, which they condition, hardly contains any of the characteristic quinone-containing secretions. We now explain this naming convention in the Material & Methods section (lines 310-311):

“The flour conditioned this way is referred to below as “*Drak* flour””

553: Baker 2016 was not a direct study of reproducibility / replication. It was a survey of researchers experiences with replication

*Authors’ response:*

Yes, this is correct. We removed the Baker reference here (lines 568-571):

“In comparison to other systematic replication studies (Errington et al., 2021; Open Science Collaboration, 2015), however, we observed higher reproducibility rates, suggesting that while reproducibility issues do exist in insect studies, they might be less pronounced than in other areas of science.“

564: "indicating that the detected treatment effects were indeed idiosyncratic to the specific laboratory"

I think this claim is somewhat misleading. The detected treatment effects in this case were idiosyncratic to the different implementations of the experiment, but based on the information available, we cannot be certain that the differences are attributable to the labs per se. For instance, it is possible that if you replicated this study in these same labs again, the results would differ among labs, but not with the same pattern.

*Authors’ response:*

Yes, we see the point and toned down our claim accordingly: “First, we found significant 'treatment-by-laboratory' interactions for A. rosae and T. castaneum experiments, indicating that the detected treatment might have been idiosyncratic to the specific laboratory, which would match the findings of Crabbe et al. (1999) [12]” (lines 578-581).

570: However, interestingly, a recent study in ecology suggests that standardizing methods does not necessarily reduce heterogeneity in results in that discipline.

Bebout, J. and Fox, J.W. (2024), Coordinated distributed experiments in ecology do not consistently reduce heterogeneity in effect size. Oikos, 2024: e10722. <https://doi.org/10.1111/oik.10722>

It might be interesting to consider why the Bebout and Fox study come to a different conclusion. I suspect it may be due to the dramatically higher true biological heterogeneity among locations in many of the systems they considered

*Authors’ response:*

We thank the reviewer for highlighting the relevant study by Bebout and Fox (2024). We agree that the difference between our conclusions and those of Bebout and Fox likely arises due to the higher biological heterogeneity in the ecological systems they investigated, compared to our controlled laboratory insect experiments. Accordingly, we now explicitly discuss this point in the revised manuscript in lines 583-589*:*

“In contrast, a recent analysis of coordinated distributed experiments in ecology suggests that reducing methodological heterogeneity across sites does not consistently reduce variation in observed effect sizes, possibly due to higher intrinsic biological variability among locations that predominates over methodological variance (Bebout & Fox, 2024). Thus, the effectiveness of standardization in minimizing between-laboratory variability may depend upon the inherent biological heterogeneity characteristic of the study system.”

599: some prior work, for instance in psychology, has found evidence that heterogeneity among results is higher when the true effect size is larger. It would be interesting to consider the applicability of this idea to your findings. See, for instance:

Klein Richard A, Vianello Michelangelo, Hasselman Fred, Adams Byron G, Adams Reginald B, Alper Sinan, Aveyard Mark, et al. Many Labs 2: Investigating Variation in Replicability Across Samples and Settings. Advances in Methods and Practices in Psychological Science. 2018;1(4):443-90. <https://doi.org/10.1177/2515245918810225>.

*Authors’ response:*

Thank you for referring us to the literature by Klein et al. (2018). While this relationship is certainly interesting, the Q statistic of heterogeneity has comparatively low power, when the number of studies in the meta analysis is small (Gavaghan et al, 2000; doi 10.1016/S0304-3959(99)00302-4). Therefore, we would rather not draw conclusions in this regard from our data. Furthermore, we fell that it might confuse readers to now discuss the concept of “heterogeneity” (of effect sizes) while simultaneously suggesting “heterogenisation” (of study designs).

621: not directly compared statistically, so maybe 'substantially' instead of 'significantly'?

*Authors’ response:*

We followed this suggestion (lines 642-646):

“The variables measured by automated tracking software, i.e., the distance moved in the sawfly larvae, yielded the most consistent results across laboratories, while the behaviour requiring manual handling in the same experiment, i.e., triggering PCI, showed substantially greater variation.”

671: as worded here, it is not clear that you are discussing statistical *significance*, as effect sizes could also be described as "overall statistical treatment". Please be explicit about when you are discussing significance testing.

*Authors’ response:*

We agree that this was not worded precisely enough and rephrased the sentence (lines 692-694):

“We were able to reproduce the overall statistical significance of the treatment effect in 83% of the replicate experiments; however, reproducing the overall effect size was successful in only 66%, while both criteria were achieved in 58% of cases.”

**Reviewer #2:**

The topic of reproducibility in science, the topic of this ms, is extremely important, and this is a solid addition to this literature. Experiments similar to what was done here have been performed for other taxa, including the landmark mouse study by Crabbe et al., but the authors claim this is the first such study for insects, and this reviewer has not seen otherwise.

The study is very comprehensive, and well executed, with a now standard 3 experimental site experimental design. In addition, the experiment involves 3 different experiments, each conducted at the 3 sites, with experimental replicates also included at each site. The findings parallel the rodent studies of this type. This is both predictable and at the same time novel, in that one might have not expected that some of the same "handling type" factors identified as being important for vertebrates would also be important for insects, given the assumption that insects are in some way less sensitive to such factors. That they are is cause for re-evaluation of experimental protocols, a provocative finding sure to spark further study and consideration.

The Introduction is excellent, identifying the important issues related to the reproducibility crisis, and how the present study relates to them. The rational for the species chosen and experiments conducted is cogent and thoughtful. The study was well designed.

The Discussion also is an excellent and thoughtful treatment of the issues and how the results of this study fit.

*Authors’ response:*

Thank you very much for the positive feedback!

I have the following relatively minor comments for possible revision.

Methods. As I recall the Crabbe et al study made a concerted effort to standardize the diets across the lab, including the sourcing of the diet components. I did not find such information in the present study. Was it done? And if not, why not?

*Authors’ response:*

We restructured the Material and Methods section and included more information on the diet (lines 180-189):

“Environmental conditions such as temperature, humidity, and light cycles were controlled and kept as consistent as possible across all laboratories (see Supplementary Tables S1-3). Diets were standardized to a large degree, but were not completely identical, as each laboratory procured the food for the insects itself by either buying, growing or collecting the necessary dietary component. In details, cabbage for A. rosae and grass blades for P. parallelus were freshly sourced locally. For T. castaneum, organic wheat flour type 550 with 5% brewer’s yeast was used. While the yeast came from the same batch, flour was bought from different distributors. All details can be found in the supplement table listing laboratory specific conditions (see Supplementary Tables S1-3).”

The red flour beetle results mirror the earlier rodent results, with variation in outcome despite genetic homogeneity due to lab-based breeding. That the variation was not attenuated relative to the wild-caught species is noteworthy. It would be good if the authors could comment on this a bit more in the Discussion.

*Authors’ response:*

While the *Tribolium* beetles come from a lab adapted population, we know from microsatellite data that they possess much larger genetic diversity than other standard laboratory stocks of the same species and thus more resemble a natural population (unpublished data). However, because we are lacking robust and detailed information on the genetic background of the beetle population, we would rather not expand our discussion on this aspect.

One possible source of variation could be the condition of the individuals based on prenatal environmental influences. Perhaps individuals came from the same populations but were of different generation? This would provide a source of variation not included in the experimental paradigm. This could be minimized by sample size, unless there are cohort differences. It would be good for the authors to comment on this possibility, even if they do not consider it to be a possible source of variation. The statistical models do not seem to consider the possibility of intrinsic differences in physiology and behavior.

*Authors’ response:*

We agree that cohort effects are a likely source of variation and thus suggest to even explicitly heterogenize across different cohorts/generations to potentially increase reproducibility (lines 721-722: “For example, one could imagine introducing biological variation by using insect populations from multiple geographic locations or from different cohorts or generations”).

We now also added more details on prenatal environmental influences as a source of variability in our study in lines 607-610:

“Apart from that, differences in reproducibility might also stem from variability in parental environmental conditions. While all A. rosae individuals were taken from one generation and population, T. castaneum individuals came from different generations, and wild-caught P. parallelus very likely also belonged to different cohorts”

In some cases animals were shipped from a common source. Could there have been any variation in shipping conditions/duration? Train transport (Exp 2) unlikely but through the mail service in other experiments?

*Authors’ response:*

We tried to minimize differences in shipping conditions as much as possible. For *T. castaneum*, the parental generation was shipped to avoid direct effects of the transport on the treated animals. For the other two species, transport happened at the same time to all laboratories (see specific lab conditions table in the Supplementary Tables 1-3)

Several figure captions (e.g., Fig. 2, 4, etc.) do not explain the figure well, i.e., what the horizontal line in the bar represents, the dots—mean/median/CI? (Some or all of the data? Summarized in what way?). Other figures do a good job of this.

*Authors’ response:*

We added more information in the figure captions of Fig. 2, 4 and 5. Here exemplary the new figure caption for Figure 4:

“**Fig 4. Preference for the green background out of the two presented backgrounds (green and brown) in the different colour morphs of the meadow grasshopper *P. parallelus* (A) across all laboratories and (B) within each laboratory**. Plotted is the preference in percent to sit on the green background, calculated from all observations (8,784 positions across 185 individual grasshoppers). Data are presented as box plots showing medians, 25% and 75% percentiles (lower and upper box), and 5% and 95% percentiles (lower and upper line). Statistics: Wilcoxon signed-rank test, two-sided. Model results from the GLMM on the transformed data show the same direction of significant differences (see Table 2). The data and code needed to reproduce this Figure can be found in https://zenodo.org/records/14002690.**”**
